# Persisting Regional Disparities in Modern Contraceptive Use and Unmet Need for Contraception among Nigerian Women

**DOI:** 10.1155/2019/9103928

**Published:** 2019-02-18

**Authors:** Chao Wang, Huimin Cao

**Affiliations:** The School of Public Policy and Management, China University of Mining and Technology, Xuzhou 221116, Jiangsu, China

## Abstract

**Background:**

Evidence suggests that equitable provision of contraceptive services can help women achieve their reproductive goals and has significant impact on reducing the rates abortion and unintended pregnancy at large. However, regional disparities continue to persist on top of low family planning prevalence which is a critical public health challenge for fast growing populations like Nigeria.

**Objectives:**

The present study aimed to explore the prevalence of (1) nonuse of modern contraceptives, (2) unmet need for contraception, and (3) regional disparities in these two.

**Methods:**

The present study used cross-sectional data obtained from the Nigeria Demographic and Health Surveys conducted in 2003, 2008, and 2013. Participants were women of reproductive of age (15-49 years) regardless of marital status. Regional disparities of nonuse of modern contraceptives and unmet need were analysed by descriptive and multivariate regression methods.

**Results:**

In the pooled sample of 79,656 participants during 2003, 2008, and 2013, 88.6% reported not using any modern methods, and 13.5% reported having unmet need for contraception. The prevalence rates of nonuse were, respectively, 91.8%, 90.6%, and 88.6% and those of unmet need were 14.2%, 16.6%, and 13.5% in the years 2003, 2008, and 2013. Significant differences were observed in the odds of reporting nonuse and unmet need for contraception across the geopolitical zones.

**Conclusions:**

The rates of nonuse of contraception are remarkably high among women in Nigeria with significant disparities across the six geopolitical zones. Efforts should be made to address the regional disparities in order to achieve the goals of universal coverage of family planning services in the country.

## 1. Introduction

Modern contraceptive use has been widely acknowledged to be one of the most cost-effective strategies for promoting reproductive health and fostering socioeconomic development globally [1]. Beyond preventing unintended pregnancies and thereby reducing the risk of unsafe abortions and maternal mortality, fertility regulation enabled by modern contraceptive use also contributes significantly to increasing women's access to educational and empowerment opportunities [2]. In order to promote reproductive rights and gender equality, the need to improve uptake of modern contraceptive methods has been consistently reiterated in the last few decades [3, 4].

Worldwide, contraceptive prevalence among women married or in-union women aged 15 to 49 years increased from 55% in 1990 to 64% in 2015. However, wide variations in contraceptive use exist across countries, with developing countries lagging significantly in this regard [5]. Current estimates indicate that 214 million women in developing countries who wish to avoid pregnancy are currently not using a modern contraceptive method [6]. Women with unmet need for modern contraception account for 84% of unintended (mistimed or unwanted) pregnancies in developing countries [7]. Nonuse of modern contraceptives is highest in sub-Saharan Africa (SSA), with the region accounting for 21% of the global burden of unmet need for modern contraception. This is worrisome as 25% of unwanted pregnancies end with abortions and 3 out of 4 abortions occurring in SSA are unsafe [8, 9]. Within the background of restrictive abortion laws, suboptimal access to maternal health services, and high burden of maternal mortality in many sub-Saharan African countries including Nigeria [10], low contraceptive prevalence represents a major public health challenge in the region that requires urgent and effective solutions.

The need to ensure universal access to modern contraception is particularly acute in Nigeria, where population control and women empowerment are crucial for achieving sustainable development [10–12]. Although trends reveal that Nigerian women are increasingly participating in education and workforce, delaying marriage and childbearing, and expressing desire to space and limit childbirths in the last few decades, studies report that the total fertility rate in Nigeria has declined marginally, from 5.7 in 2003 to 5.5 in 2013 [10, 13–16]. This suggests that nonuse of modern contraceptive remains a problem in Nigeria that limits women from achieving their reproductive desires and socioeconomic aspirations. Additionally, research indicates that modern contraceptives use is lowest among women in the least developed parts of Nigeria, where early marriages and low female literacy levels are also rife and only a small proportion of women utilize maternal health care services [14–16]. Thus, regional disparities in contraceptive use result in further deprivation among women who are already grappling with multiple dimensions of health and socioeconomic disadvantage.

The Nigerian Government is partnering with donor agencies to intensify media campaigns to drive demand for contraceptives and strengthen the supply of family planning commodities at primary health care facilities at no cost to women [15, 17–20]. While it is hoped that these interventions will accelerate the achievement of the targeted 36% contraceptive prevalence rate by 2018, evidence suggests that the country has recorded marginal progress and is still a long way from this goal [14]. The current situation underscores the need to intensify efforts to increase uptake of modern contraceptives in Nigeria. However, significant progress is unlikely to occur if regional equity gaps are not effectively addressed. Although contraceptive prevalence in Nigeria has received a lot of focus, the persistence of regional disparities in uptake of modern contraceptives appears to have garnered insufficient attention. This paper is aimed at the exploring trends in regional disparities in the prevalence of nonuse of modern contraceptive methods and unmet need for modern contraception in Nigeria.

## 2. Methods

### 2.1. Data Collection

Data for this study were derived from three rounds of Demographic and Health Survey in Nigeria conducted in 2003, 2008, and 2013. In Nigeria, the surveys are implemented by the National Population Commission (NPC) with the financial and technical assistance from ICF International provisioned through the USAID-funded MEASURE DHS program. DHS surveys are nationally representative that collect information on a wide range of public health related topics such as anthropometric, demographic, socioeconomic, family planning, and domestic violence to name a few. The survey covered men and women aged between 15 and 49 years and under-5 children residing in noninstitutional settings [21]. For sampling, a three-staged stratified cluster design was employed which was based on a list enumeration areas (EAs) from the 2006 Population Census of the Federal Republic of Nigeria. EAs are systematically selected units from the localities, which constitute the local government areas (LGAs). LGAs are subdivisions of each of the 36 administrative states (including the Federal Capital Territory called Abuja) and classified under six developmental zones in the country. EAs were used to form the survey clusters called primary sampling units. A more detailed version of the survey was published elsewhere [22].

### 2.2. Study Variables

Outcome variable was prevalence of modern contraceptive use and unmet need for contraception. Unmet need for contraception was categorized dichotomously as ‘Yes' if the respondent reported having unmet need and ‘No' if reported otherwise.

Independent variables of primary interest were regional disparity in modern contraceptive use. NDHS survey provided two such indicators: (1) type of place of residence (urban/rural) and (2) geopolitical region (north-central/north-east/north-west/south-east/south-south/south-west).

To adjust the analysis for potential confounders, the following variables were included based on their theoretical relevance to the outcome variable:

Age: 15-19/20-24/25-29/30-34/35-39/40-44/45-49; marital status: in union/widowed/other; religious affiliation: Christian/Islam, others; educational attainment: nil/primary/secondary/higher; employed: yes/no; wealth index: poorest/poorer/middle/richer/richest; sex of household head: male/female; parity: nullipara/primipara/multipara; history of abortion: no/yes.

For the calculation household wealth status, instead of direct income the volume of durable goods (e.g., TV, radio, and bicycle) possessed by the household and housing quality (e.g., type of floor, wall, and roof) are taken into consideration. Each item is assigned a factor score generated through principal component analysis (PCA) which are then summed and standardized for the households. These standardized scores place the households in a continuous scale based on relative wealth scores. The scores are thus obtained from a continuous scale and subsequently categorized into quintiles to rank the household as poorest/poorer/middle/richer/richest to richest [23].

### 2.3. Data Analysis

All analyses were performed with SPSS Version 24 for Windows. To adjust for the cluster sampling techniques of the surveys we used complex survey module for all analysis by accounting for primary sampling units, sample strata, and sample weight. Following that, descriptive analyses were carried out to calculate the prevalence rates of contraceptive use and of unmet need. Chi-square tests were performed to examine the bivariate association between the two outcome variables and the explanatory variables. Variables that were found to be significant at alpha 5% were entered into regression analysis. Two sets of binary logistic regression models were carried out to calculate the odds ratios of the association between contraceptive nonuse and unmet need for contraception while adjusting for the sociodemographic variables. The level of significance was set at alpha 5% for the regression models.

#### 2.3.1. Ethical Consideration

Before taking part in the interview, all participants gave informed consent to the surveyors. DHS surveys are also approved by ICF International as well as an Institutional Review Board (IRB) in the host country to make sure that the protocols are in compliance with the U.S. Department of Health and Human Services regulations for the protection of human subjects.

## 3. Result

### 3.1. Descriptive Statistics

The basic sociodemographic profile of the sample population for last three DHS surveys conducted in Nigeria was presented in Table 1. Rate of participation was highest for 2013 with most of the women belonging to the youngest age group of 15 to 19 years and averaging below 30, originating from the north-west developmental region, being rural residents, currently in union, followers of Christian faith, and having no formal education. More than half of the women were currently unemployed and were living in the richer to richest households (except for in 2008). Percentages of female headed households are quite low with a slow but steady increase over the last decade (16.3% in 2003 versus 18.3% in 2013). The prevalence of nulliparity in 2013 (29.5%) was slightly higher than in 2008 (28.9), however lower compared to 2003 level (32.5%), while that of multiparity rose from 56% in 2003 to 60.2% in 2008, but fell marginally to 59.2% in 2013. The prevalence of abortion has declined by 3.9% during the same period (14.5% in 2003 versus 10.6% in 2013).

### 3.2. Prevalence of Nonuse of Modern Contraceptives and Unmet Need for Contraception

The prevalence rates of nonuse were, respectively, 91.8%, 90.6%, and 88.6, and those of unmet need were 14.2%, 16.6%, and 13.5 in the years 2003, 2008, and 2013.

**Figure 1:**
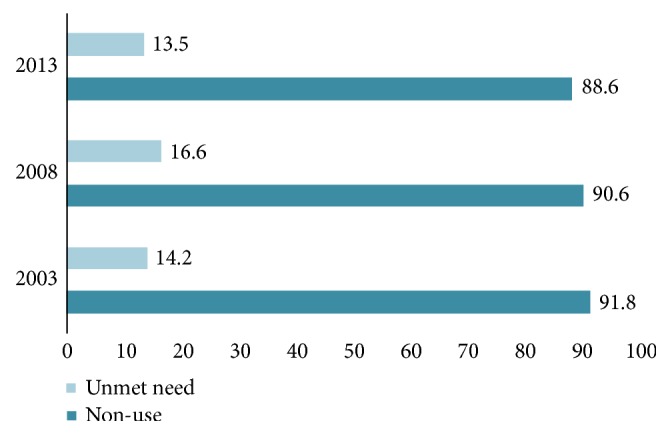
Prevalence of contraceptive nonuse and unmet need stratified by survey years. NDHS 2003-13.

Figure 1 illustrates that prevalence of both nonuse of modern contraceptives and unmet need have been decreasing albeit slowly.

**Figure 2:**
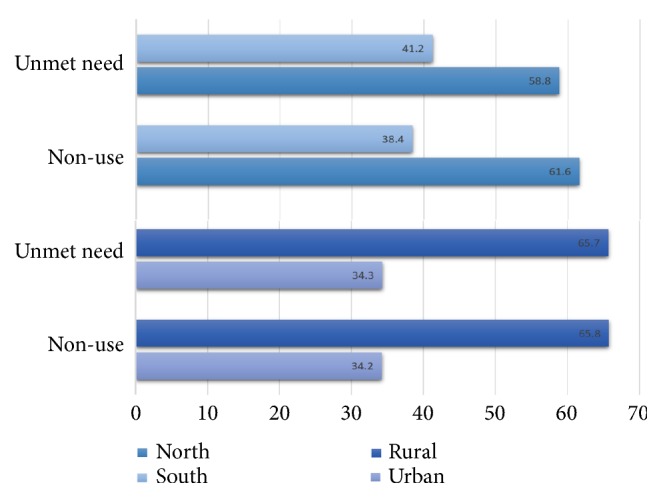
North-south and urban-rural disparities in the overall prevalence of contraceptive nonuse and unmet need. NDHS 2003-13.

Figure 2 shows that the overall prevalence of both nonuse and unmet need were higher in Northern compared with southern regions and higher in rural compared with urban areas.

At the second step of the analysis we performed Chi-square tests to assess the bivariate relationships between the outcome variables and the explanatory variables.  Table 2 indicates that the prevalence of both nonuse of modern contraceptive methods and unmet need for contraception decreased with increasing age, except for unmet need in 2013. Significant variations were observed across six geopolitical regions and between urban and rural areas too as women from the more developed regions such as south-south and south-west were less likely to report nonuse and unmet need compared to the least developed ones such as north-east and north-west. Marital status and religious affiliation did not appear to have any significant association with either of the outcome variables (except for nonuse in 2013). Women's educational attainment and household wealth status were also found to be significantly associated with nonuse of modern contraceptives and unmet need. Women who were multiparous and from male-headed households were significantly more likely to report nonuse in the latest survey.

### 3.3. Multivariate Association between Nonuse of Modern Contraceptives and the Geographic Parameters

Table 3 shows the adjusted associations between nonuse of modern contraceptives and unmet need for contraception with developmental regions and urbanicity. Results indicate that residing in the less developed regions (compared to the most developed one) was associated with significantly higher odds of reporting nonuse and unmet need. However, the associations appeared to be more consistent in the case of nonuse for all the regions except South-south in the years 2003 and 2008. Compared with south-south, the odds of nonuse were 3.6 times as high [AOR=3.598; 95%CI= 2.503-5.171] in the north-east which is by far the least developed region in the country. Importantly, the odds of nonuse in 2003 in the north-central compared with south-west region were 41% [AOR=1.417; 95%CI=1.025-1.959] higher which decreased to be 25% [AOR=1.249; 95%CI=1.088-1.434] in 2013. Similar decreasing pattern was noticed in all other regions as well except for south-south. However, the scenario was contrary in the case of unmet need as the odds increased between 2003 and 2013 in the north-central and north-east regions. With regard to types of residence, urban women in 2008 were 15% [AOR=0.847; 95%=0.748-0.960] less likely to report nonuse compared with their rural counterparts.

## 4. Discussion

Main findings: in the present cross-sectional study based on data derived from Nigeria Demographic and Health Survey, we aimed to measure the prevalence nonuse of modern contraceptives methods and unmet need for contraception and the existence of any regional disparities in their prevalence rates. Several important findings emerged from this analysis that merit special attention. The overall prevalence of nonuse was remarkably high among the participants as close to 90% were not using any modern method. The percentage has been declining however at a marginal rate since 2003. In 2013, the prevalence of nonuse has decreased by a mere 3 percentage points during the past decade. Regarding unmet need for contraception, overall about one in seven women reported currently having any unmet need. In 2013, the prevalence was 13.5%, indicating that little progress has been achieved over the last decade (14.2% in 2003).

Levels of nonuse and unmet need for contraception varied significantly greatly across the sociodemographic subgroups. In the north, the prevalence of nonuse and unmet need was near about 1 in 3 compared with 1 in 2 in the South. Similar disparities were observed between urban and rural settings as well. These north-south and urban-rural variations in the prevalence rates were confirmed in the multivariable regression analysis. In general, the odds of nonuse and unmet need were higher in the northern parts and rural areas. The exact causes of these variations are difficult to explain in light of the present findings. However, it is assumable that these disparities are the reflection of the deeply ingrained socioeconomic inequalities across different geopolitical regions in the country as depicted by national and internally renowned news media [14, 24–26].

## 5. Comparison with Previous Findings

Rate of contraceptive use rate in Africa is historically low (13% in 1990s) [27]. In a recent study based on Demographic and Health Surveys from 18 countries sub-Saharan Africa it was reported that the average rate of nonuse was 92.4% among the population [23]. However, the study was limited to respondents aged 15-19 years only. Country representative evidences on family planning researches are still scarce in Nigeria. However, findings from studies at regional level also indicate a suboptimal use of contraception among adolescent and adult Nigerian women. A cohort study in South East Nigeria reported the prevalence of any type of contraceptive use to be 28.3% and that of modern methods to be 16.3% [28]. Another study involving participants aged between 15 and 24 years found that the prevalence of ever use was 11.1% and that of current use 7.3% [29].

Regarding unmet need, one study in Cross-River State of Nigeria found that unmet need for modern contraception was as high as 49% among women seeking antiretroviral therapy (ART), 75% among those seeking HIV counselling and testing (HCT), and 32% among those seeking prevention-of-mother-to-child-transmission of HIV (PMTCT) services (Okigbo CC, 2015). These rates are higher than the ones observed in the present study. For studies with dissimilar sample and methodological approaches, it is recommended to interpret the comparisons in light of definitions and type of unmet need for contraception used. For instance, the unmet for birth spacing can differ significantly among married and unmarried women and for those seeking the service for birth spacing rather birth limiting.

Regarding unmet need, one study in Cross-River State of Nigeria found that unmet need for modern contraception was as high as 49% among women seeking antiretroviral therapy (ART), 75% among those seeking HIV counselling and testing (HCT), and 32% among those seeking prevention-of-mother-to-child-transmission of HIV (PMTCT) services [22]. These rates are higher than the ones observed in the present study. For studies with dissimilar sample and methodological approaches, it is recommended to interpret the comparisons in light of definitions and type of unmet need for contraception used. For instance, the unmet for birth spacing can differ significantly among married and unmarried women and for those seeking the service for birth spacing rather birth limiting [30, 31].

Current evidence based on regional disparities in contraceptive use and unmet need is insufficient and inclusive to make critical comparisons. Although concrete statistics are not available, several of the previous studies on family planning and maternal healthcare programs in Nigeria have mentioned the existence of regional disparities [32, 33]. A common finding is that residents of the Southern regional and urban setting are more likely to adhere to family planning services and less likely to have unmet needs of contraception. Although, the findings of the present study cannot confirm any causal effect, given the present scenario it is however assumable that addressing the north-south and urban-rural gap holds certain potential to promote contraceptive use and other family planning services in the country.

## 6. Recommendation for Policy Action and Future Researches

The findings of the present analysis have important implications for policy making. With one of the fastest growing population and having high fertility rate, Nigerian Government has shown strong commitments to control population growth and improve reproductive healthcare services. Nigeria also ranks high among the countries with high maternal mortality rates which makes it an urgent imperative to increase research and development investments on family planning and other core maternal healthcare services. Evidence shows that the socioeconomic gap in the use of maternal healthcare services has been decreasing slowly; however, significant regional disparities continue to persist in the provision and use of family planning services. Our findings further support the need for strengthening political efforts to resolve the geopolitical issues for promoting the use of family planning services in the country. Identifying the causes of regional discrimination was not within the scope of the present study, and therefore the needs for further researches to investigate the sources of disparities and approaches for to resolving those are warranted.

## 7. Strengths and Limitations

The study has several strength and limitations to report. Firstly, the sample size was large and pooled that allowed measuring the overall prevalence of contraceptive nonuse and unmet need for last three surveys. Data were analysed using appropriate techniques for cluster samples. The findings were reported in light of the existing evidences to provide a comparative understanding of the scenario in Nigeria. However, the comparison of the findings with previous studies should be done keeping in mind the methodological approaches used to assess modern contraceptive and unmet need. The limitation includes cross-sectional nature of the surveys that prevent making any causal inference about the association. As the data were secondary, we had no control over the selection and measurement of the variables. Lastly, information on contraceptive use was self-reported; hence the chances of reporting bias should not be ignored while interpreting the findings.

## 8. Conclusions

The present study provides an update on the prevalence of nonuse and unmet need for contraception among adult women in Nigeria. Based on the findings, we conclude that the prevalence of nonuse of modern contraception was strikingly high with a considerably large proportion of women facing unmet need for contraception. For both of the indicators statistically significant disparities were observed across regions in the prevalence rates. Promoting modern contraceptive use and addressing unmet need are of paramount importance to reduce pregnancy related morbidity and mortalities and improve reproductive well-being among women. These arguments suggest that strengthening national policy efforts and family planning programs should be regarded a public health priority and must address the underlying sociopolitical barriers to equitable provision of family planning services in the population.

## Figures and Tables

**Table 1:** Sociodemographic characteristics of the participants. NDHS 2003-13.

**Variables**	**Pooled**	**2003**	**2008**	**2013**
	N=79,656 (%)	N=7,568 (%)	N=33,140 (%)	N=38,948 (%)

**Age (Mean/SD)**	28.68/9.59	27.95/9.57	28.62/9.48	28.86/9.68
15-19	16203 (20.3)	1744 (23.0)	6554 (19.8)	7905 (20.3)
20-24	14256 (17.9)	1461 (19.3)	6081 (18.3)	6714 (17.2)
25-29	14662 (18.4)	1355 (17.9)	6270 (18.9)	7037 (18.1)
30-34	10838 (13.6)	939 (12.4)	4526 (13.7)	5373 (13.8)
35-39	9339 (11.7)	790 (10.4)	3848 (11.6)	4701 (12.1)
40-44	7331 (9.2)	676 (8.9)	2992 (9.0)	3663 (9.4)
45-49	7027 (8.8)	603 (8.0)	2869 (8.7)	3555 (9.1)
**Geopolitical region **				
North-central	13819 (17.3)	1252 (16.5)	6316 (19.1)	6251 (16.0)
North-east	14197 (17.8)	1409 (18.6)	6158 (18.6)	6630 (17.0)
North-west	18633 (23.4)	1755 (23.2)	7205 (21.7)	9673 (24.8)
South-east	9190 (11.5)	1076 (14.2)	3652 (11.0)	4462 (11.5)
South-south	11792 (14.8)	936 (12.4)	4798 (14.5)	6058 (15.6)
South-west	12025 (15.1)	1140 (15.1)	5011 (15.1)	5874 (15.1)
**Type of place of residence **				
Urban	28993 (36.4)	3034 (40.1)	10414 (31.4)	15545 (39.9)
Rural	50663 (63.6)	4534 (59.9)	22726 (68.6)	23403 (60.1)
**Marital status **				
In union	54676 (68.6)	4966 (65.6)	23307 (70.3)	26403 (67.8)
Widowed/other	24,980 (31.4)	2602 (34.4)	9833 (29.7)	12545 (32.2)
**Religious affiliation **				
Christian	38,155 (47.9)	3700 (48.9)	17085 (51.6)	19838 (50.9)
Islam	34,253 (43.0)	2451 (32.4)	15293 (46.1)	18578 (47.7)
Other	7248 (9.1)	1417 (18.7)	762 (2.3)	532 (1.4)
**Educational attainment **				
Nil	29805 (37.4)	2965 (39.2)	13100 (39.5)	13740 (35.3)
Primary	15317 (19.2)	1662 (22.0)	6551 (19.8)	7104 (18.2)
Secondary	27724 (34.8)	2454 (32.4)	10863 (32.8)	14407 (37.0)
Higher	6810 (8.5)	487 (6.4)	2626 (7.9)	3697 (9.5)
**Employed **				
Yes	32134 (40.3)	3369 (44.5)	13823 (41.7)	14942 (38.4)
No	47522 (59.7)	4199 (55.5)	19317 (58.3)	24006 (61.6)
**Wealth index **				
Poorest	15280 (19.2)	1467 (19.4)	7211 (21.8)	6602 (17.0)
Poorer	15662 (19.7)	1389 (18.4)	6758 (20.4)	7515 (19.3)
Middle	16046 (20.1)	1500 (19.8)	6545 (19.7)	8001 (20.5)
Richer	16478 (20.7)	1530 (20.2)	6498 (19.6)	8450 (21.7)
Richest	16190 (20.3)	1682 (22.2)	6128 (18.5)	8380 (21.5)
**Sex of household head**				
Male	65737 (82.5)	6332 (83.7)	27567 (83.2)	31838 (81.7)
Female	13919 (17.5)	1236 (16.3)	5573 (16.8)	7110 (18.3)
**Parity**				
Nullipara	23526 (29.5)	2457 (32.5)	9572 (28.9)	11497 (29.5)
Primipara	8880 (11.1)	871 (11.5)	3610 (10.9)	4399 (11.3)
Multipara	47250 (59.3)	4240 (56.0)	19958 (60.2)	23052 (59.2)
**History of abortion **				
No	70798 (88.9)	6468 (85.5)	29544 (89.1)	34834 (89.4)
Yes	8858 (11.1)	1100 (14.5)	3596 (10.9)	4114 (10.6)

**Table 2:** Trends in nonuse and unmet need for modern contraceptive methods among women in Nigeria. 2003-2013 DHS.

	**Non-use (88.6**%**)**				**Unmet need (13.5**%**)**			
	**2003**	**2008**	**2013**	**Pooled**	**2003**	**2008**	**2013**	**Pooled**
**Age**								
15-19	24.2	20.9	21.7	21.6	9.5	10.5	20.3	18.7
20-24	18.8	17.9	16.8	17.5	17.7	16.8	17.1	17.2
25-29	17.4	18.7	17.8	18.1	19.1	20.4	18.2	18.4
30-34	12.3	13.3	13.5	13.3	17.1	15.8	13.2	14.2
35-39	10.1	11.3	11.7	11.4	13.6	14.8	12.3	12.4
40-44	8.9	8.9	9.1	9.0	13.6	12.1	10.1	9.8
45-49	8.3	8.9	9.4	9.1	9.5	9.6	8.8	9.2
p-value	0.028	<0.0001	<0.0001	<0.0001	<0.0001	0.019	ns	<0.0001
**Geopolitical region**								
North-central	16.3	18.8	15.7	17.0	21.0	17.4	17.9	16.1
North-east	19.9	19.8	18.6	19.2	22.4	18.6	18.3	17.2
North-west	24.6	23.4	27.2	25.4	17.6	25.6	23.0	25.0
South-east	14.2	11.0	11.1	11.4	11.9	8.2	10.8	11.1
South-south	11.4	13.3	14.3	13.6	15.5	16.1	16.1	15.6
South-west	13.6	13.7	13.2	13.4	11.6	14.1	13.8	15.0
p-value	ns	<0.0001	0.014	<0.0001	<0.0001	<0.0001	<0.001	<0.0001
**Type of place of residence **								
Urban	38.4	29.5	37.5	34.2	38.8	27.8	38.5	38.4
Rural	61.6	70.5	62.5	65.8	61.2	72.2	61.5	61.6
p-value	0.043	<0.0001	<0.0001	<0.0001	<0.0001	<0.0001	0.025	<0.0001
**Marital status **								
In union	63.8	70.3	67.7	68.4	76.3	74.8	68.8	68.9
Widow	36.2	29.7	32.3	31.6	23.7	25.2	31.2	31.1
/other								
p-value	ns	ns	ns	ns	ns	ns	ns	ns
**Religious affiliation **								
Christian	31.2	48.9	47.3	44.6	32.2	47.4	50.9	50.4
Islam	17.6	48.7	51.3	45.7	20.8	50.1	47.5	46.9
Other	51.2	2.4	1.5	9.7	47.0	2.5	1.6	2.7
p-value	ns	ns	<0.0001	<0.0001	ns	.932	ns	<0.0001
**Education**								
Nil	42.0	42.5	39.0	40.8	40.9	45.3	36.4	36.6
Primary	22.0	19.7	18.1	19.1	26.2	22.6	17.8	19.1
Secondary	30.7	31.3	35.0	33.0	28.4	26.9	36.4	35.5
Higher	5.3	6.5	7.9	7.1	4.5	5.2	9.5	8.8
p-value	<0.0001	<0.0001	<0.0001	<0.0001	0.047	0.001	ns	<0.0001
**Employed **								
Yes	45.7	42.9	39.7	41.6	33.6	37.0	37.8	38.1
No	54.3	57.1	60.3	58.4	66.4	63.0	62.2	61.9
p-value	ns	ns	<0.0001	<0.0001	.024	ns	ns	<0.0001
**Wealth index **								
Poorest	20.5	23.4	18.9	20.9	20.5	22.5	16.7	17.8
Poorer	19.3	21.5	20.8	21.0	17.5	22.3	19.2	19.7
Middle	20.5	20.0	20.7	20.4	20.6	20.9	21.2	20.5
Richer	19.9	18.6	20.6	19.7	21.4	20.0	22.9	21.3
Richest	19.9	16.4	18.9	18.0	19.9	14.4	19.9	20.7
p-value	.039	<0.0001	<0.0001	<0.0001	ns	.03	0.012	<0.0001
**Sex of household head**								
Male	83.1	83.2	82.7	83.0	85.2	87.2	82.1	82.5
Female	16.9	16.8	17.3	17.0	14.8	12.8	17.9	17.5
p-value	ns	ns	<0.0001	<0.0001	ns	ns	ns	<0.0001
**Parity**								
Nullipara	35.8	28.8	29.2	29.7	37.9	25.3	29.7	28.1
Primipara	12.7	10.9	11.5	11.4	13.4	11.0	10.9	11.0
Multipara	51.6	60.3	59.3	59.0	48.7	63.7	59.4	60.9
p-value	ns	ns	<0.0001	<0.0001	ns	ns	ns	<0.0001

N.B. ns=not significant. p-values are from *X*^2^-tests.

**Table 3:** Odds ratios of association between nonuse of contraception and unmet need with regional variables. NDHS 2003-13.

	**Non-use**				**Unmet need**			
	**OR (95**%**CI)**				**OR (95**%**CI)**			
	**2003**	**2008**	**2013**	**2003-13**	**2003**	**2008**	**2013**	**2003-13**

**Geopolitical region**								
South-west (ref)	**1**	**1**	**1**	**1**	**1**	**1**	**1**	**1**
North-central	**1.417 (1.025-1.959)**	**1.181 (1.007-1.385)**	**1.249 (1.088-1.434)**	**1.559 (1.454, 1.672)**	**0.582 (0.439-0.771)**	1.111 (0.952-1.296)	**1.221 (1.090-1.368)**	**1.096 (1.046, 1.149)**
North-east	**3.598 (2.503-5.171)**	**2.083 (1.629-2.663)**	**2.654(2.113-3.333)**	**1.820 (1.732, 1.912)**	**0.602 (0.442-0.819)**	1.063 (0.893-1.264)	**1.171 (1.027-1.334)**	**1.357 (1.298, 1.419)**
North-west	**2.319 (1.588-3.387)**	**2.797 (2.033-3.848)**	**2.264(1.467-3.494)**	**1.590 (1.483, 1.706)**	1.067 (0.778-1.463)	0.908 (0.765-1.078)	0.969 (0.847-1.109)	1.026 (0.973, 1.082)
South-east	**2.201 (1.585-3.056)**	**2.205 (1.851-2.627)**	**1.831 (1.612-2.080)**	0.926 (0.804, 1.250)	0.810 (0.603-1.112)	**1.351 (1.141-1.600)**	1.026 (0.905-1.164)	**1.282 (1.221, 1.347)**
South-south	1.109 (0.817-1.505)	1.076 (0.926-1.249)	**1.272 (1.124-1.439)**	0.855 (0.787, 1.029)	**0.520 (0.382-0.708)**	0.928 (0.710-1.365)	1.128 (0.999-1.275)	**1.066 (1.014, 1.121)**
**Type of place of residence **								
Rural (ref)	**1**	**1**	**1**	**1**	**1**	**1**	**1**	**1**
Urban	0.814 (0.643-1.031)	**0.847 (0.748-0.960)**	0.913 (0.820-1.016)	**1.820 (1.73-1.912)**	1.057 (0.887-1.260)	1.090 (0.981-1.212)	0.971 (0.893-1.056)	**1.096 (1.046, 1.149)**

N.B. Significant associations (p<0.05) are shown in bold. Regression models are adjusted for age, religious affiliation, education, employment, wealth index, sex of household head, and parity.

## Data Availability

All data used in this study are available through the DHS program website to the registered users: dhsprogram.com.
